# OXIDANT/ANTIOXIDANT STATUS IN OBESE ADOLESCENT FEMALES WITH ACNE VULGARIS

**DOI:** 10.4103/0019-5154.48984

**Published:** 2009

**Authors:** Khalid O Abulnaja

**Affiliations:** *From the Department of Biochemistry, Faculty of Science, King Abdulaziz University, Jeddah, Saudi Arabia*

**Keywords:** *Acne vulgaris*, *adolescent*, *obese females*, *oxidative stress*

## Abstract

**Background and Objectives:**

Acne vulgaris is a distressing skin condition, which can carry with it significant psychological disability. Oxidant/antioxidant imbalance leads to increased production of free radicals, that cause many diseases. Some nutrients, along with systemic oxidative stress, have been implicated in acne vulgaris. The goal of the present study was to assess oxidant and antioxidant status in correlation with the incidence of acne vulgaris in adolescent obese females.

**Materials and Methods:**

A total of 60 adolescent females (age 16-22 years) were divided into four groups (15 each) as follows: The first included obese females with acne; the second included obese females without acne; the third included non obese with acne and the fourth included non obese without acne. Fasting serum Malondialdehyde (MDA), β-carotene, and Vitamins A, E, and C were measured. In addition, platelet monoamineoxidase (MAO), and erythrocyte catechol-o-methyltransferase (COMT) activities were determined.

**Results:**

It was found that serum MDA was statistically significantly decreased in obese and non obese subjects with acne, as compared to those without acne (*P*<0.05, *P*<0.001) respectively. In contrast, the levels of β-carotene, vitamins A, E and C and the activity of MAO were significantly decreased in the obese and non obese with acne, as against the obese and non obese without acne.

**Interpretation:**

In obese subjects, increased fat content facilitates free radical production and lipid peroxidation, as indicated by increased MDA level, which is scavenged by the antioxidant vitamins. The decreased activity of MAO may be inhibited by free radicals and this causes psychological depression in adolescents. However there were non significant changes in the activity of COMT among the studied groups.

**Conclusion:**

The nutritional factors and a weakened antioxidant defense system may interplay, to increase the risk of psychological sequelae in acne vulgaris.

## Introduction

Acne vulgaris is a distressing skin condition which can carry with it significant psychological disability. Patients with acne are more likely to experience anger and are at increased risk of depression, anxiety and suicidal ideation. Certain nutrients that have been implicated as influencing the pathophysiology of acne have also been identified as important mediators of human cognition, behavior and emotions.[1] Zinc, folic acid, selenium, chromium and ω-3 fatty acids are all examples of nutrients that have been shown to influence depression, anger and/or anxiety. The same nutrients, along with systemic oxidative stress, have been implicated in acne vulgaris and may interplay to increase the risk of psychological sequelae in acne vulgaris.[2]

Lipid peroxidation and the value of systemic antioxidants have been reported to be of value in acne.[3] Important antioxidant enzymes, including glutathione peroxidase and super oxide dismutase, have been reported to be significantly lower in the blood of acne patients.[4] Higher levels of oxidative stress have been documented in social anxiety, a condition more common in those with acne. While depression and anxiety can certainly influence oxidative stress, the oxidative stress theory of depression/anxiety also dictates that some of the symptoms may be directly mediated by free radical damage to lipid components of the central nervous system.[5] In keeping with this theory, experimental data shows anxiolytic and antidepressant effects of antioxidant rich polyphenols, green tea, turmeric and berries.[6]

Ascorbate has a great reducing potential and reacts with many reactive oxygen and nitrogen species *in vitro*. Ascorbate effectively quenches singlet oxygen, superoxide, hydroxyl and water soluble peroxyl radicals, and hypochlorous acid.[7] The reaction between ascorbate and those reactive species generates a one-electron oxidation product (ascorbyl radical) that can be oxidized further to result in dehydroascorbate. Cellular reducing molecules (e.g., nicotinamide adenine dinucleotide reduced form [NADH], nicotinamide adenine dinucleotide phosphate reduced form [NADPH], and glutathione) can reversibly reduce one- and two-electron oxidation products to ultimately regenerate ascorbate.[8] In addition, ascorbate can recycle the vitamin E radical (tocopheroxyl), thereby forming the ascorbyl radical, which can be regenerated.[9]

Monoamines (MAOs) are integral proteins of the outer mitochondrial membranes in various cells (both neuronal and non neuronal in the central nervous system (CNS) and peripheral organs), where they oxidatively deaminate biogenic and xenobiotic amines.[10] In the CNS, they play a pathophysiological role by indirectly generating cytotoxic free radicals during aging and in neurodegenerative diseases.

The therapeutic potential of selective reversible MAO inhibitors lies in their ability not only to increase the biological half-life of monoamine transmitters (symptomatic effects) but also to slow down the process of neurodegeneration (neuroprotective effects).[11] Monoamine oxidase B(MAO) and catechol-o-methyltransferase (COMT) are pivotal enzymes in the catabolism of several neurotransmitters. The MAO-B and COMT activity can be reliably measured in human platelets and erythrocytes, respectively.[12]

This study aimed to assess the oxidative stress status in obese adolescent females with acne and its correlation with psychological depression during this period.

## Materials and Methods

This study was conducted on 60 adolescent females, of ages between 16 and 22 years (mean,18.5 years), divided into 4 four groups (each of 15) as follows: The first included obese females with severe acne. The second included obese females without acne, the third included non obese with acne and the fourth included non obese without acne.

Mild acne was defined as up to 10 lesions, moderate acne between 11 and 25 lesions, and severe acne as more than 25 lesions. Obesity was defined by body mass index (BMI) > 27 kg/m^2^, and waist to hip ratio (WHR) > 0.8, according to Garrow and Webster.[13]

All the cases were selected from among the outpatients of the dermatology unit of the University Hospital, King Abdulaziz University (Jeddah, Saudi Arabia). They were submitted to clinical examination (non diabetic, free from any neuroendocrine disorders, no history of hypertension, liver, kidney or heart disease) and not under any treatment.

Ten ml of fasting blood samples were withdrawn from all the cases and were divided into three portions, the first for serum separation, the second for platelet separation and the third for erythrocyte separation. Serum Malondialdehyde was determined according to Satoh.[14] Serum vitamin A, E and β-carotene levels were determined by high performance liquid chromatography (HPLC), according to Lee,[15] and vitamin C according to McCormick and Wright.[16]

Platelet collection and determination of MAO activity were carried out by the fluorimetric method of McEntire.[17] Platelet MAO activity was calculated in terms of nmol/h/mg protein. The COMT activity in erythrocytes was determined by the fluorimetric method of Axerod and Tomchick.[18] The COMT activity in erythrocytes was calculated in terms of pmol/h/mg hemoglobin.

### Statistical analysis

The results were expressed as mean ±SD. Statistical comparisons were made using the student t-test, nonparametric chi square, and Kruskal-Wallis tests. *P* value less than 0.05 was accepted as the significance level.

## Results

The sociodemographic data in Table 1 revealed that, the age of the adolescent females selected in this study was between 16 and 22 years. There was a significant elevation in body mass index (BMI) in obese females with or without acne, as compared with non obese females with or without acne (*P* < 0.05).

**Table 1:** Sociodemographic characteristics of obese and non obese females with or without acne (mean ± SD)

Groups parameters	Obese		Non obese	
			
	(with acne)	(without acne)	(with acne)	(without acne)
Age Mean ± SD	18 ± 1.3	20 ± 1.9	17 ± 1.4	18 ± 1.62
t_1_ (p)	NS	-	NS	-
t_2_ (p)	NS	NS	-	-
BMI	30 ± 2.1	29 ± 1.7	26 ± 2.1	25 ± 1.9
Mean ± SD				
t_1_ (p)	NS	-	NS	-
t_2_ (p)	1.3	p < 0.05	-	-
	(p < 0.05)			

t_1_ (p): t test and significance between obese female with acne vs without acne and non obese with acne vs without acne; t_2_ (p): t test and significance between obese female with acne or without acne vs non obese with or without acne; NS: non significant

Serum malondialdehyde (MDA) and antioxidant vitamin levels are presented in Table 2. It was found that the Serum MDA level was very significantly elevated in obese females with acne, seen as against the obese without acne (*P* < 0.001); in the non obese with acne seen against the non obese without acne (*P* < 0.05); and, in the obese with acne, seen against the non obese with acne (*P* < 0.001).

**Table 2:** Serum malondialdehyde and antioxidant vitamins levels in obese and non obese females with or without acne (mean ± SD)

Groups parameters	Obese		Non obese	
		
	(with acne)	(without acne)	(with acne)	(without acne)
MDA (mmol/L) Mean ± SD	2.93 ± 0.81	0.93 ± 0.36	1.16 ± 0.41	0.83 ± 0.29
t_1_ (p)	3.50* (*P* < 0.001)	-	*P* < 0.05	-
t_2_ (p)	2.03(*P* < 0.001)	NS	-	-
β-Carotene (μg/dl) Mean ± SD	0.11 ± 0.006	0.32 ± 0.05	0.14 ± 0.29	0.38 ± 0.07
t_1_ (p)	7.65 (*P* < 0.05)	-	*P* < 0.01	-
t_2_ (p)	NS	NS	-	-
Vitamin A (mmol/L) Mean± SD	33.2 ± 7.4	42.7 ± 8.1	39 ± 6.6	64.9 ± 10.1
t_1_ (p)	6.27 (*P* < 0.05)	-	*P* < 0.01	-
t_2_ (p)	NS	*P* <0.01	-	-
Vitamin E (μmol/L) Mean ± SD	2.91 ± 0.7	5.64 ± 0.9	3.9 ± 1.0	6.9 ± 1.5
t_1_ (p)	3.80 (*P* < 0.05)	-	<0.01	-
t_2_ (p)	2.02 (*P* < 0.001)	NS	-	-
Vitamin C (mmol/L) Mean ± SD	22.8 ± 9.4	42.9 ± 9.8	31 ± 8.5	46.1 ± 12.3
t_1_ (p)	4.65 (*P* < 0.01)	-	*P* < 0.01	-
t_2_ (p)	<0.05	NS	-	-

t_1_ (p): t test and significance between obese female with acne vs without acne and non obese with acne vs without acne; t_2_ (p): t test and significance between obese female with acne or without acne vs non obese with or without acne; NS: non significant,

* very significant, MDA: malondialdehyde

In contrast, the levels of serum beta-carotene, vitamins A, E and C were statistically significantly decreased in the obese with acne, as compared with the obese without acne (*P* < 0.05, < 0.01, < 0.01 and < 0.001) respectively; and, decreased in the non obese with acne, as compared with the non obese without acne (*P* < 0.001, < 0.01, < 0.01 and 0.01) respectively. Comparing the obese with acne with the non obese with acne, it was revealed that there was a significant decrease in the level of vitamin C, in obese females (*P* < 0.05); there were no changes in the levels of other vitamins.

Table 3 revealed that the activity of platelet MAO was statistically significantly decreased in the obese with acne vis-à-vis the obese without acne (*P* < 0.001) and in the non obese with acne vis-à-vis the non obese without acne (*P* < 0.05). However, the decrease in the obese was more than that in the non obese. The activity of erythrocyte COMT showed nonsignificant changes among the obese with or without acne vis-à-vis the non obese with or without acne.

**Table 3:** The activities of platelet monoamine oxidase (MAO) and erythrocyte catechol-o-methyltransferase (COMT) in obese and non obese females with or without acne (mean ± SD)

Groups parameters	Obese		Non obese	
		
	(with acne)	(without acne)	(with acne)	(without acne)
Platelet MAO (μmol/L)	3.7 ± 1.3	10 ± 4.7	7.5 ± 3.4	12.61 ± 2.3
t_1_ (p)	7.07 (*P* < 0.001)	-	3.16 (*P* < 0.05)	-
t_2_ (p)	NS	NS	-	-
Erythrocyte	1.61 ± 0.29	1.57 ± 0.3	1.46 ± 20.3	1.53 ± 0.44
COMT (μmol/h/RBCs)				
t_1_ (p)	NS	-	NS	-
t_2_ (p)	NS	NS	-	-

t_1_ (p): t test and significance between obese female with acne vs without acne and non obese with acne vs without acne; t_2_ (p): t test and significance between obese female with acne or without acne vs non obese with or without acne; NS: non significant

A positive correlation was found between the elevation in MDA level and the decreased the activity of MAO (r = 0.61).

## Discussion

For decades. lipid peroxidation and the value of systemic antioxidants have been reported to be of value in acne. Only recently have investigators used sophisticated techniques to establish that not only is oxidative stress and lipid peroxidation a local issue but also that these patients are under systemic oxidative stress. Antioxidant enzymes, including glutathione peroxidase and super oxide dismutase have been reported to be significantly lower in the blood of acne patients. Higher levels of oxidative stress have been documented in social anxiety,[19] a condition more common in those with acne. While depression and anxiety can certainly influence oxidative stress, the oxidative stress theory of depression/anxiety also dictates that some of the symptoms may be directly mediated by free radical damage to lipid components of the central nervous system.[20]

The goal of this study is to interpret the causes of depression in adolescent females in correlation to the acne vulgaris. It was found that the Serum MDA level was significantly elevated, while the levels of β-carotene and vitamins A, E and C were significantly decreased in the obese with acne, as compared with the obese without acne; and, in the non obese with acne, as compared with the non obese without acne.

Recently, it was reported that in 100 patients with acne (vs. matched controls), the systemic levels of vitamins A and E were much lower overall; also, the lower the levels of vitamins A and E, the more severe was the acne.[21] As with most chronic medical conditions characterized by both oxidative stress and inflammation, it is likely that the blood levels of antioxidants are used up more readily in those with acne, because there is a greater demand to deal with free radicals. In the context of the psychological sequelae in acne, we make reference to the oxidative stress-acne connection, because it has been shown that depressive symptoms are independently correlated with oxidative stress.[22]

We suspect that in acne increased risk of psychological sequelae would be low levels of certain nutrients, which can further increase that risk. Prospective trials, which evaluate mood and emotions and yet are also inclusive of nutritional assessments and related blood markers, would help to shed light on the complex relationships between diet, the risk of acne and its individual mental health consequences.

Ascorbate, not tocopherol, normalizes epidermal lipid profiles (in particular glucosphingolipids and ceramides) in the reconstructed epidermis. Until recently, its instability in cosmetic forms had limited its use. Ascorbic acid is commonly used in a number of cosmetic products that claim to protect the skin from environmental insults and photo aging. However, in many, but not all cases, ascorbic acid is present at a low concentration, is not stable, or is not delivered efficiently to the skin. In addition, vitamin E exhibits scavenging activities against a wide spectrum of free radicals including singlet oxygen, superoxide, and hydroxyl radicals. *In vitro*, tocotrienol scavenges peroxyl radicals and is recycled more efficiently than tocopherol.[23]

It is our contention that nutritional factors may be setting the stage for a higher risk of depression, anxiety and other emotional symptoms related to acne. It is likely that a significant group of teenagers and young adults are already on the pathway to an increased risk of psychological impairment before the acne even becomes evident. Emerging studies with matched controls shows that acne patients are more likely to consume carbohydrates, fast foods, soft drinks and sweets.[24]

Regardless of the controversial impact that such dietary choices may have on clinical acne, it is possible that nutritional voids in subgroups of acne may influence the emotional realm. Research also shows that the psychological sequelae in acne can be long term, and in some cases, resistant even after clinical improvement in acne. To test this hypothesis it would be interesting to evaluate young children for signs of depression and anxiety and later determine who might go on to experience acne. Investigations of this nature have already determined that depressive and anxious symptoms in early school years can predict later overweight and obesity when followed up.[25]

The monoamine oxidases and flavoenzymes play an important role in the catabolism of biogenic and xenobiotic organisms by oxidative deamination. In the human brain, MAO-A preferentially deaminates adrenaline; MAO-B and COMT activity in humans can be reliably measured in peripheral tissues easily accessible for sampling. The MAO activity in platelets has been shown to be an accurate model of neuronal MAO activity.[26]

The activity of MAO was significantly decreased in obese females with acne, compared with those without acne, This inhibition increases monoamine levels in those subjects and this causes psychological depression. However, non significant change was observed in the activity of COMT among the studied groups. The COMT in human erythrocytes reflects activity in the liver, kidney and lung. The autoxidation and monoamine oxidase (MAO)-mediated reaction cause a continuous production of hydroxyl radical (·OH), which is further enhanced by the presence of iron. Consequently monoamines have been shown to be a double-edged sword, because they display antioxidant properties in relation to lipid peroxidation and also exhibit pro-oxidant properties by causing both generation of ·OH and oxidation of mitochondrial proteins.[27]

It is recommended that body weight must be reduced and there should be increased intake of nutrients rich in antioxidant activity and of vitamins, to overcome this psychological depression period.
